# Niche Markets

**DOI:** 10.1371/journal.pbio.0020147

**Published:** 2004-05-11

**Authors:** Peter Manning, H. Charles J Godfray

## Abstract

Is evolutionary theory is incomplete and are we failing to understand phenomena as disparate as ecosystem development and the interplay of genes and culture in shaping human evolution?

This book, the latest in the excellent Monographs in Population Biology series from Princeton University Press, is a work of advocacy in which the authors argue that evolutionary theory is incomplete and that, in consequence, we are failing fully to understand phenomena as disparate as ecosystem development and the interplay of genes and culture in shaping human evolution. What we are missing, they argue, is an appreciation of niche construction, the process by which an organism modifies the abiotic and biotic environment in which it is subject to natural selection. The authors' major assertion is that the importance of niche construction is so great that it should be regarded “after natural selection, as a second major participant in evolution” and that it is “not just an important addition to evolutionary theory” but “requires a reformulation of evolutionary theory”. Bold claims indeed.[Fig pbio-0020147-g001]

**Figure pbio-0020147-g001:**
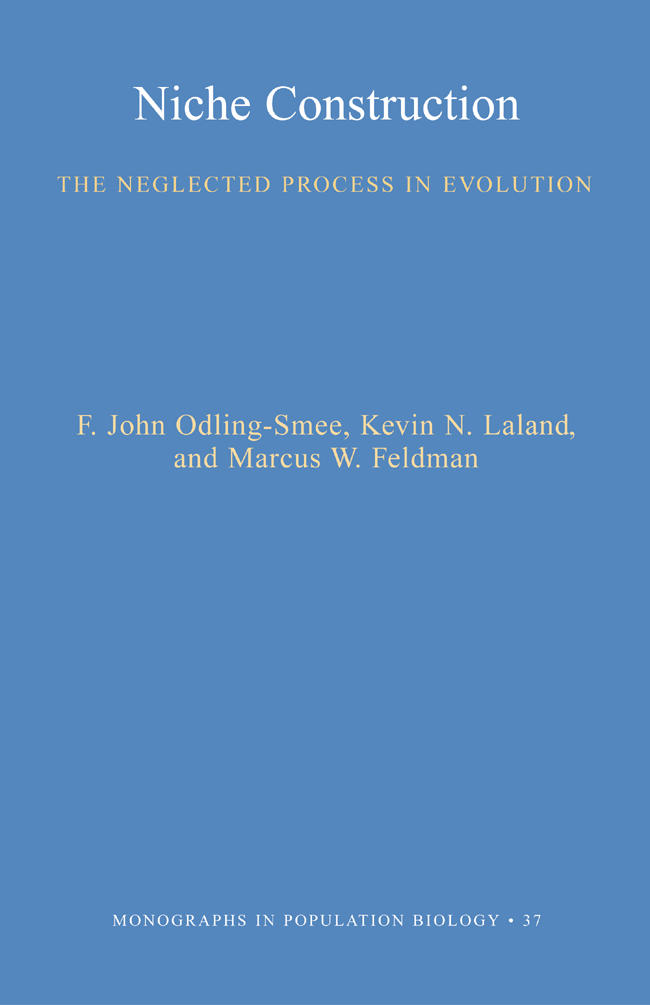


After introducing the conceptual framework Odling-Smee et al. set out a series of arguments to support this position, the first of these being the empirical case for the existence of niche construction. Niche construction, as broadly interpreted here, is everywhere. Animals build nests, burrows, and protective cases and so alter the environment they experience in a way that may select for further adaptations. The changes caused by some animal species, such as beavers and earthworms, are of a sufficient magnitude that the environment experienced by a host of other species is affected. Many plant species also modify the environment they experience by generating organic litter; influencing hydrological and biogeochemical cycles; affecting temperature, humidity, and light regimes; and, over the longer term, determining the make up of the atmosphere. Decomposer and chemoautotrophic microorganisms similarly influence biogeochemical transformations, while parasitic species can manipulate the behaviour and internal environment of the hosts they infect. Perhaps less obvious examples of niche construction are the many types of migration and cultural evolution that, like physical transformations, cause the organism's descendants to experience a different selective environment.

After this broad, accessible survey, the authors change key rather abruptly and explore two-locus, frequency-dependent population genetics. The novelty here is that selection on one locus depends on the history of gene frequencies at the other, “niche construction”, locus. In an extension, gene frequencies at one locus affect an environmental variable with its own dynamics that in turn influences the second locus. As one would guess, the models display a range of potentially interesting dynamics, though generalisations and broad conclusions are sparse. We guess the aim of the chapter is to illustrate that environmental feedbacks can be potent and general agents of evolutionary change, but the restriction of the theory to such a narrow model, with very technical explanation, risks losing the few readers who we suspect will stay the course (did we really need a rederivation for haplodiploids?).

Perhaps aware of the dangers of getting bogged down in detail, the argument then moves to proving a case for the universality of niche construction. Invoking the second law of thermodynamics and Maxwell's Demon, the authors lead us through a challenging thesis that concludes that the persistence of life on earth requires both natural selection and niche construction, thereby justifying some of the bold claims for their new theory. We think they are technically correct, but we are concerned that the demonstration of the inescapability of niche construction, as defined here, does not guarantee that it will actually tell us new and important things about the world, as the theory of natural selection has.

The remainder of the book explores the implications of niche-construction thinking for evolutionary biology, ecology, and the human sciences, and in our view is the most successful part. Though it is rare for the authors to offer new analysis and insight, their sideways look at many issues from the niche-construction viewpoint often offers interesting new angles on old problems, and suggests new avenues of enquiry that may be the book's greatest legacy. A good example of this is their convincing and timely argument that a more explicit recognition of evolution's role in environmental feedbacks will help to unify population/community ecology and ecosystem science.

The chief argument for the prosecution is that niche construction is common but not pervasive, and that wherever ecologists and evolutionists have found interesting examples of it, they have developed appropriate theory and concepts to understand its ramifications. For example, some of the clearest examples of niche construction occur in plant succession where, as F.E. Clements realised nearly a hundred years ago, early-succession plants frequently modify their environment in ways that allow other species to replace the pioneers. Interestingly, the strict Clementsian theory of facilitation, niche construction *avant la lettre*, has given way to a more pluralistic theory of succession. It is a great pity that the authors give so little space to plants and plant ecology, as it here that some of the finest examples of niche construction are found, as well as the best-developed conceptual framework for studying the roles of environmental feedback.

Other areas where biologists have well-developed theories of the influences and impacts of niche construction include co-evolutionary theory, where the environmental feedbacks are largely biotic, and Dawkins' theory of the extended phenotype. Very close to some of the arguments discussed here, and generously acknowledged, is the idea developed by Jones, Lawton, and colleagues of ecosystem engineers, species that have a major impact on the abiotic environment experienced by a large number of species.

A major strength of the book is that it reveals common processes and patterns underlying disparate biology in consistently interesting ways. Its chief contribution is thus not to tell us new things about how nature works but to link together many different aspects of ecology under an umbrella of theory that may in the future lead to new insights. Do they deliver on their grand claims? Time will tell, but our view is that they don't. They engagingly admit that for their project to succeed the new theory must earn its keep by producing significant new biology—something which has yet to occur. However, the great need for the biological and human sciences to integrate across subdisciplines, as the authors bravely attempt here, makes this a hugely worthwhile book. Its breadth of scope and its boldness in creating syntheses have resulted in a stimulating and challenging read.

